# Complete adnexal torsion of a large dermoid ovarian cyst with ovarian and tubal preservation in a 17-year-old patient: a case report

**DOI:** 10.11604/pamj.2026.53.50.40749

**Published:** 2026-02-03

**Authors:** Anna Thanasa, Efthymia Thanasa, Evangelos Kamaretsos, Ioannis Paraoulakis, Vasiliki Grapsidi, Emmanouil Xydias, Evangelos-Ektoras Gerokostas, Ioannis-Rafail Antoniou, Gerasimos Kontogeorgis, Athanasios Chasiotis, Apostolos Ziogas, Ioannis Thanasas

**Affiliations:** 1 Department of Health Sciences, Medical School, Aristotle University of Thessaloniki, Thessaloniki, Greece,; 2 Department of Obstetrics and Gynecology, General Hospital of Trikala, Trikala, Greece,; 3 Department of Obstetrics and Gynecology, EmbryoClinic IVF, Thessaloniki, Greece,; 4 Department of Obstetrics and Gynecology, Limassol General Hospital, Limassol, Cyprus,; 5 Department of Health Sciences, Medical School, University of Thessaly, Larissa, Greece

**Keywords:** Dermoid ovarian cyst, adnexal torsion, ultrasound, cystectomy, case report

## Abstract

Dermoid cyst or mature cystic teratoma is the most common ovarian germ cell tumor. Clinical diagnosis is not easy. The acute abdominal pain associated with ovarian dermoid cysts usually involves adnexal torsion. Preoperative diagnosis of adnexal torsion with dermoid ovarian cyst can be based on pelvic imaging combined with clinical findings. Treatment is surgical and should be applied without delay. Cystectomy is the treatment of choice for the management of dermoid ovarian cysts that have undergone torsion and maintain adequate blood supply. Our case report concerns the emergency surgical intervention of a patient with right adnexal torsion and the presence of a large dermoid cyst. The patient came to the Emergency Department of our hospital with symptoms of an acute abdomen. Ultrasound confirmed the diagnosis of adnexal torsion with the presence of a mature cystic ovarian teratoma, and laparotomy with cystectomy was performed. Histological examination of the surgical specimen confirmed the diagnosis of twisted ovarian dermoid cyst. At this point, we must emphasize the crucial contribution of transabdominal pelvic ultrasound in the management of mature cystic ovarian teratomas accompanied by complete adnexal torsion. Early diagnosis and immediate surgical intervention in young patients who wish to preserve ovarian function in order to achieve future pregnancy are of major importance.

## Introduction

Dermoid cyst, or mature cystic teratoma, is the most common ovarian germ cell tumor. It was first described as a nosological entity by Johannes Scultetus in 1659 (Arooj *et al*.) [1]. The ovarian dermoid cyst in its typical form contains skin, hair, nervous tissue, and sebaceous glands. It is a benign tumor that affects approximately 20% of all ovarian tumors. It usually occurs in women of reproductive age (<40 years), but it also occurs less frequently in perimenopausal and postmenopausal women. The risk of malignant transformation is rare. It is estimated to affect 1.5%-2% of cases and concerns mostly elderly patients or large tumors [2]. More often than malignant transformation, mature ovarian cystic teratoma may undergo spontaneous rupture, or, even more commonly, adnexal torsion may occur. Torsion of adnexa with an ovarian dermoid cyst appears more often in young patients and does not seem to be related to the mean diameter of the cyst [3]. In this paper, after the case report, a brief literature review of the early diagnostic and therapeutic approach to torsion of the adnexa with an ovarian dermoid cyst is attempted.

## Patient and observation

**Patient information:** a 17-year-old patient came to the emergency department of our hospital complaining about acute, intense abdominal pain, accompanied by multiple episodes of vomiting. The onset of symptoms was reported approximately two hours ago. Our patient had no history of sexual intercourse. The menstrual cycle was normal. The menstrual blood loss was normal. Personal and family medical history was free.

**Clinical findings:** a bimanual pelvic examination was not performed. The patient did not have an active sexual life. On palpation of the abdomen, pain was mainly located in the right iliac fossa with radiation throughout the abdomen. There were signs of peritoneal irritation. The patient had no fever. Blood pressure and heart rate were normal.

**Figure 1:**
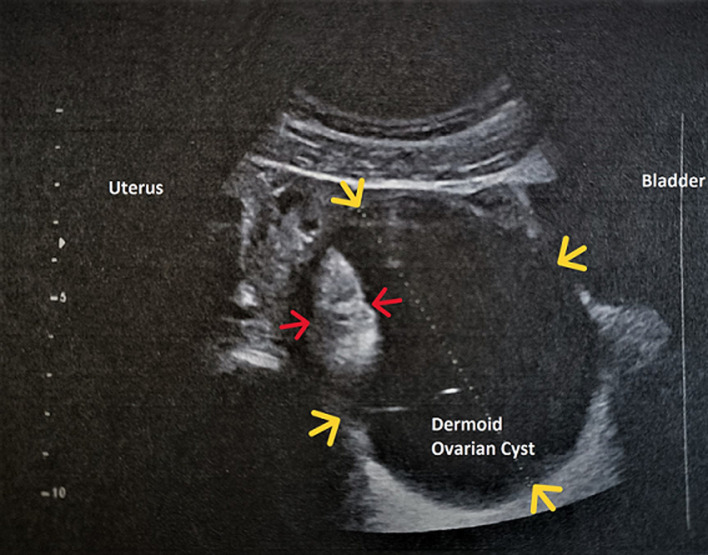
transabdominal ultrasound imaging of a twisted dermoid ovarian cyst (yellow arrows) with the typical presence of fat within it (red arrows)

**Diagnostic assessment:** transabdominal ultrasound in the anatomical position of the right ovary revealed a unilocular, well-circumscribed cystic mass with a maximum diameter of about 105 mm. Solid components were observed within the cystic mass (Figure 1). The left ovary was normal. No pathology from the uterus was found. Urgent blood tests of our patient revealed Ht 38.6%, Hb 13.4gr/dl, PLT 234x10^3^/ml, WBC 10.38x10^3^/ml, NEUT 78.6%. C-reactive protein, coagulation, and biochemical tests were within normal range.

**Figure 2:**
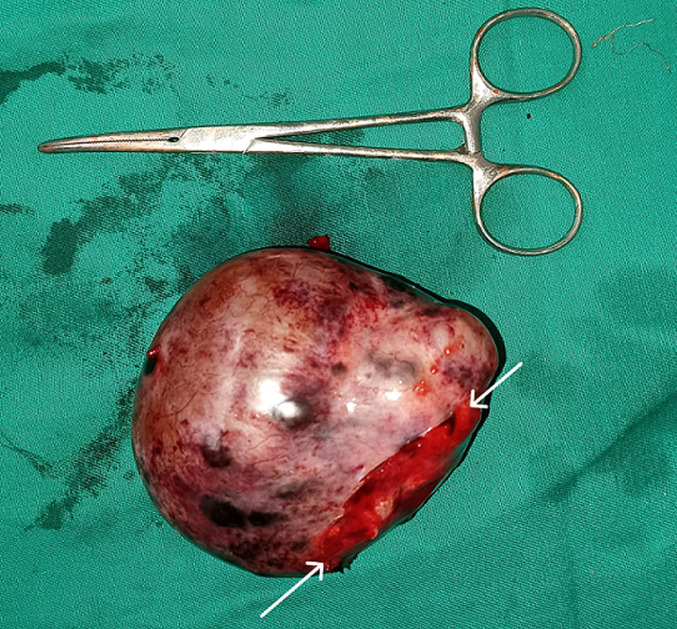
surgical specimen of a twisted dermoid ovarian cyst after cystectomy with sufficient blood supply at the area of resection from the ovary (white arrows)

**Figure 3:**
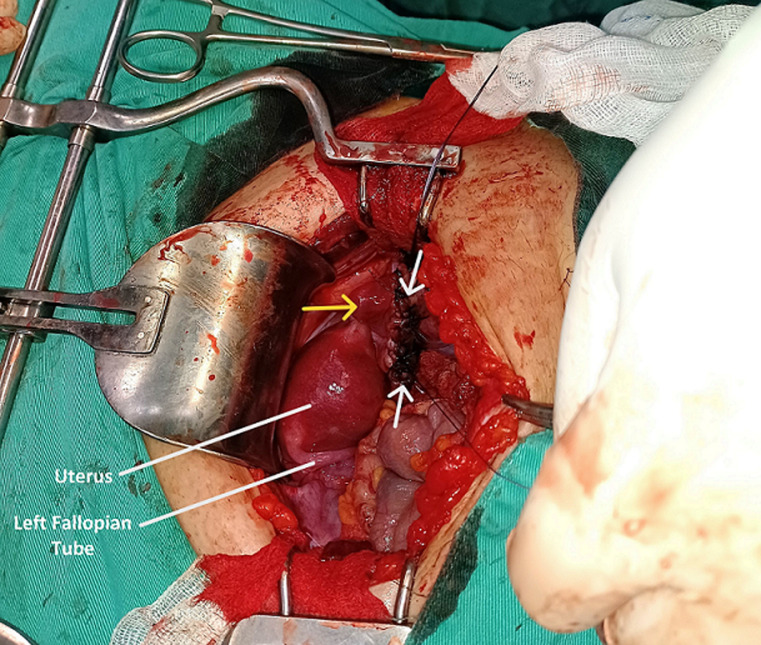
intraoperative image of suturing the remaining ovarian tissue (white arrows) and fallopian tube with good perfusion and mild edema (yellow arrow)

**Figure 4:**
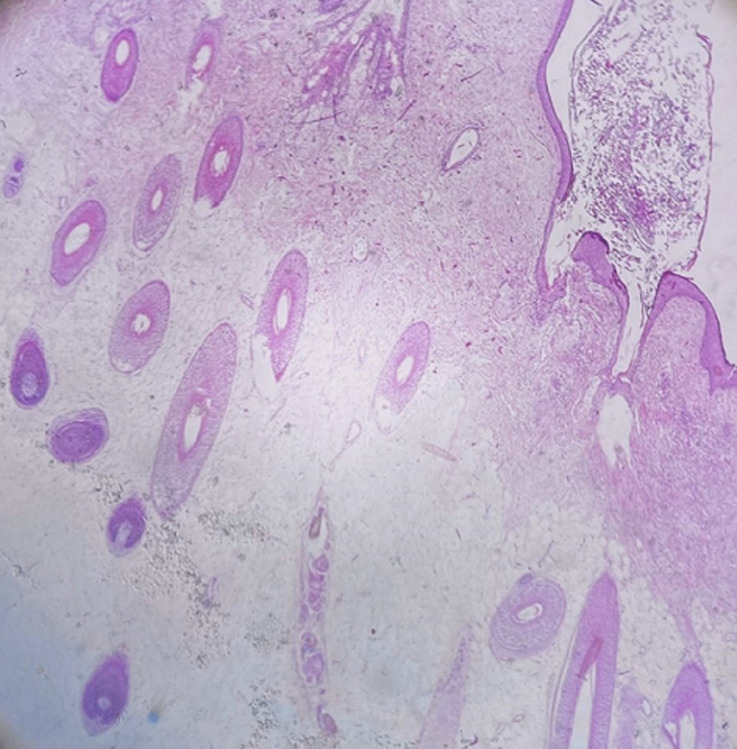
histological image of a twisted dermoid ovarian cyst: imaging of the cyst wall with hair follicles and skin is evident

**Figure 5:**
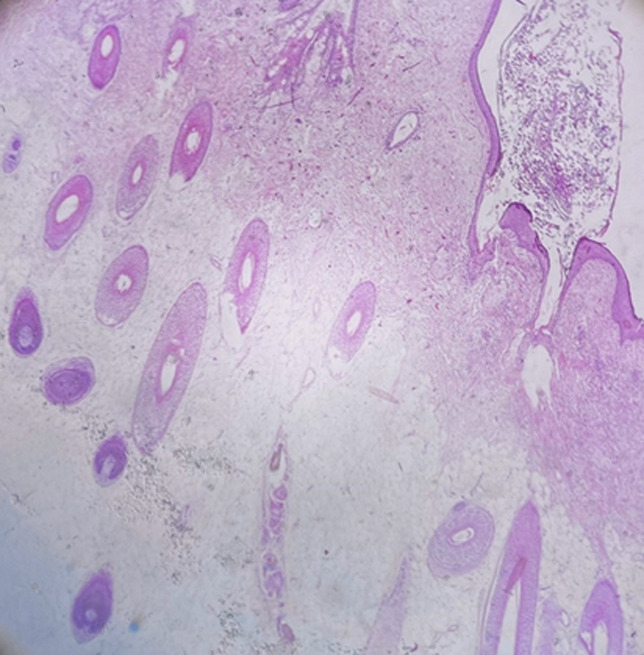
histological image of a twisted dermoid ovarian cyst: imaging of nervous tissue with a choroidal plexus is evident

**Therapeutic intervention:** the severe symptomatology, the suspected complete adnexal torsion, and the young age of the patient led to the decision to perform an emergency laparotomy. The patient herself and her family were consulted about the necessity of emergency surgery. The laparoscopic approach was not available at our hospital. Intraoperatively, the presence of a large twisted ovarian mass, without signs of total necrosis, was found in the right parametrium. After immediate detorsion of the twisted adnexa and revascularization of the affected adnexa, a cystectomy with ovarian tissue preservation was performed (Figure 2). Histological examination of the surgical specimen (Figure 3) confirmed the diagnosis of a twisted ovarian dermoid cyst (Figure 4, Figure 5).

**Follow-up and outcomes:** on the fourth postoperative day, the patient was discharged from the clinic. Two months after surgery, on ultrasound, the right ovary was imaged as normal. Six months after surgery, the ovary remained normal with the presence of follicles, with no signs of recurrence of mature cystic teratoma. The postoperative follow-up of the patient continues at regular intervals at the gynecology outpatient clinic of the hospital.

**Patient perspective:** the patient was satisfied with the treatment she received.

**Informed consent:** it was obtained and the anonymity of the patient was maintained for confidentiality.

## Discussion

Ovarian dermoid cysts are slow-growing tumors that usually remain asymptomatic for a long time and are diagnosed incidentally during pelvic imaging. In our patient, the diagnosis of dermoid cyst was made after the sudden onset of acute abdominal pain and vomiting. The acute abdominal pain associated with ovarian dermoid cysts may rarely involve rupture, more commonly may indicate adnexal torsion with evident clinical signs and laboratory markers of acute inflammation. In our patient, complete torsion of the ovarian pedicle was contra distinctly, not accompanied by an increase in inflammatory markers. White blood cell count, neutrophil percent, and quantification of C-reactive protein were within normal range. This could probably be attributed to the brief period between the onset of symptoms and the arrival of the patient at the hospital and the sampling of blood for testing. Blood-based biomarkers of inflammation are estimated to be statistically significantly higher in patients with adnexal torsion [4]. Also, liver dysfunction (elevated levels of aspartate transaminase and alanine aminotransferase) may be associated with torsion of mature cystic ovarian teratoma [5]. The differential diagnosis between spontaneous rupture of mature cystic ovarian teratoma and adnexal torsion with an ovarian dermoid cyst is challenging in routine clinical practice and requires punctual diagnostic and therapeutic management.

Early preoperative diagnosis of adnexal torsion with an ovarian dermoid cyst is very important. For optimal management of the condition, a diagnostic strategy that includes multiple imaging techniques such as ultrasound, computed tomography, and magnetic resonance imaging is necessary. Ultrasound is currently the most common and widely available imaging for the preoperative evaluation of ovarian dermoid cysts. It is currently considered that the acute symptomatology of patients in the emergency department combined with ultrasound findings contributes significantly to accurate diagnosis of adnexal torsion with the presence of a mature ovarian cystic teratoma. In our patient, the characteristic ultrasound feature of solid components within the well-circumscribed cystic mass (Figure 1), combined with acute abdominal pain, multiple vomiting, and the young age of the patient, established the diagnosis of twisted mature ovarian cystic teratoma. Early diagnosis, based only on clinical and ultrasound findings, contributed significantly to the timely surgery and the preservation of the ovary and fallopian tube in our patient. On ultrasound imaging, the presence of a dermoid plug of variable size, with one or more highly echogenic nodules within the cyst or atypical only within the wall of the cystic mass, advocates the diagnosis of mature cystic teratoma. Also, a fat-fluid level or a distinct echogenic focus indicating the presence of an ectopic tooth is a strong ultrasound feature for the diagnosis of teratoma. Ultrasound diagnosis of ovarian dermoid cysts can be based on transabdominal ultrasound with pelvic imaging [6]. In our patient, the diagnosis was based exclusively on transabdominal ultrasound. No transvaginal ultrasound was performed because the patient was a virgin.

Computed tomography (CT) scan should be limited, especially in young patients, due to the use of ionizing radiation. Computed tomography scans in most patients with dermoid ovarian cysts reveal a pelvic mass with calcification and sebaceous material [7]. It is currently considered that CT combined with blood markers (CRP - C-reactive protein, cancer antigen 125 - CA125, carbohydrate antigen 19-9 - CA19-9, squamous cell carcinoma antigen - SCC) could contribute significantly to the preoperative diagnosis of adnexal torsion with a mature cystic ovarian teratoma. Patients with ruptured dermoid cysts have significantly higher levels of serum CRP, CA125, CA19-9 and SCC compared to those patients with adnexal torsion with dermoid cyst. Also, the contribution of CT scans in the diagnostic approach of patients with chronic rupture of ovarian dermoid cysts is important. In these cases, multiple scattered peritoneal and mesenteric masses containing fat and calcification are demonstrated and can differentiate the condition from the torsion of the mature ovarian cystic teratoma [7]. Magnetic resonance imaging (MRI) can be used to manage patients with particular diagnostic difficulties. Characteristic of ovarian teratoma on MRI is the typical, but rare, appearance of “sack of marbles” corresponding to free-floating intracystic globules of sebum/fat of mixed content [8]. In any case of complete adnexal torsion with a mature cystic ovarian teratoma, especially when it concerns young patients who wish to preserve fertility, treatment should be surgical and applied immediately, without delay. In our patient, timely, accurate diagnosis led to the decision to perform surgery immediately. The early therapeutic intervention resulted in the removal of only the dermoid cyst (cystectomy) and the preservation of the fallopian tube and ovary. Cystectomy by laparoscopy or laparotomy, as a minimally invasive treatment, is the treatment of choice for the management of ovarian dermoid cysts that have undergone torsion and retain adequate blood supply without the presence of irreversible necrotic lesions. Accurate intraoperative assessment of ovarian ischemia in case of adnexal torsion with mature cystic teratoma is of great clinical significance in order to avoid unnecessary unilateral oophorectomy or adnexectomy [9]. In our patient, the waiting time given intraoperatively after detorsion of the twisted adnexa helped in reperfusion of the affected tissue and the correct decision to preserve the fallopian tube and ovary. Laparoscopic ovarian cystectomy performed within-endobag seems to be absolutely indicated in patients with ruptured dermoid cyst or in patients with large teratomas, in order to reduce the risk of rupture and spillage of the cyst contents into the peritoneal cavity [10]. In our patient, laparoscopy was not feasible because it is not available in our hospital. Also, a recent cohort study shows that barbed suture versus conventional suture is more effective in sparing the ovary and preserving ovarian function after resection of mature cystic ovarian teratoma by laparo-endoscopic single-site surgery [11].

## Conclusion

The adnexal torsion with the presence of a dermoid cyst in the ipsilateral ovary is an urgent clinical condition. Transabdominal ultrasound imaging of the pelvis in conjunction with clinical findings can be of significant help in the correct preoperative diagnosis of this condition. Its inclusion in the differential diagnosis of patients presenting with sudden acute abdominal pain and the presence of a pelvic mass should be a main concern of the modern gynecologist. Early diagnosis and treatment are essential, especially in young patients who wish to preserve ovarian function.
